# Stable Isotope Ratio Analysis for the Discrimination of the Geographic Origin of Rice (*Oryza sativa* L.)

**DOI:** 10.3390/foods14183163

**Published:** 2025-09-11

**Authors:** Anna-Akrivi Thomatou, Eleni C. Mazarakioti, Anastasios Zotos, Achilleas Kontogeorgos, Angelos Patakas, Athanasios Ladavos

**Affiliations:** 1 Department of Food Science & Technology, University of Patras, 30100 Agrinio, Greece; e.mazarakioti@ac.upatras.gr (E.C.M.); apatakas@upatras.gr (A.P.); 2 Department of Sustainable Agriculture, University of Patras, 30100 Agrinio, Greece; azotos@upatras.gr; 3 Department of Agriculture, International Hellenic University, 57001 Thessaloniki, Greece; akontoge@ihu.gr

**Keywords:** geographical origin, authenticity, rice, isotope ratio mass spectrometry (IRMS)

## Abstract

Discrimination of geographical origin can satisfy the demand for food authenticity while decreasing the risk of adulteration in high-quality food products. Rice is among the most important cultivated crops worldwide, providing food for more than half of the Earth’s population. The aim of this study is to discriminate the geographical origin of rice cultivated in three regions: Agrinio (Western Greece), Serres, and Chalastra (Central Macedonia). In total, 120 samples were collected from Agrinio and 160 samples from Serres during each cultivation period (2021 and 2022), as well as 90 samples from Chalastra (sampling periods 2023 and 2024). The isotope ratios of the light elements (C, N, and S) were measured using isotope ratio mass spectrometry (IRMS), and the results obtained were analyzed using chemometric techniques, one-way ANOVA, Multivariate Analysis of Variance (MANOVA), and a decision tree algorithm. The mean values of delta permille (*δ* ‰) calculated from the one-way ANOVA were *δ*^15^N = 4.64‰, *δ*^13^C = −26.8‰, and *δ*^34^S = 3.62 for rice from Agrinio; *δ*^15^N = 5.34‰, *δ*^13^C = −26.1‰, and *δ*^34^S = −0.903 for rice from Serres; and *δ*^15^N = 5.90‰, *δ*^13^C = −28‰, and *δ*^34^S = 4.01 for rice from Chalastra. The decision tree algorithm achieved high accuracy (91.9%), sensitivity (from 86.1% for Agrinio to 97.9% for Serres), and specificity. The results obtained from the decision tree algorithm show that this method could be used to discriminate rice cultivars from the three Greek regions.

## 1. Introduction

The origin of food is a matter of great concern for consumers, who show considerable interest in food authentication. Consumers spend considerable amounts on agricultural products that meet certain specifications of quality and safety; however, at the same time, high prices can easily enhance fraudulent activities with products of uncertain origin. Demand for food authentication has led the EU to highlight the significance of the geographical origin of food by introducing regulations for “food and feed traceability” to define food origin (Traceability Regulation 178/2002/EC). Food authenticity is the process by which foodstuffs are checked for their origin, the accuracy of their label description, and whether they are adulterated or mislabeled and meet certain quality standards or regulations [1,2].

Food safety improvement and quality assurance are crucial issues for producers and traders at all levels of food production [3,4]. Therefore, tools that validate food authenticity, such as the geographical origin of food, are essential [5]. Accurate identification of the geographical origin of agricultural products to prevent counterfeiting and adulteration is of great concern to both consumers and traders.

Rice (*Oryza sativa* L.) is one of the most significant agricultural products as it constitutes a staple carbohydrate source for over half of the world’s population. Moreover, it provides protein, energy, vitamins, and essential elements for human beings [6]. Rice also possesses a range of antioxidative, anti-inflammatory, immune-stimulating, anti-allergic, and anticarcinogenic properties because it is a rich source of many bioactive compounds such as phenolics, flavonoids, and sterol derivatives [7,8]. More specifically, antioxidants, such as phytochemicals, minerals, vitamins, and phenolic compounds, including tannins, phenolic acids, and flavonoids, suspend oxidative reactions, remove harmful free radicals, and protect humans from cancer and heart disease [9,10]. According to Okonogi et al. (2018) [11], different rice varieties exhibit varying antioxidant and anti-inflammatory activities, as studied in comparisons between white rice and pigmented rice. Discrimination of rice variety and growing region is essential to prevent mislabeling and adulteration [10,12]. Furthermore, geographical origin determination is not easy when cultivation areas are geographically close to each other. Therefore, validation of rice authenticity is difficult and can result in mislabeling and adulteration of rice with inferior-quality products [13].

Various methods have been applied for the determination of the geographical origin of foodstuffs, and numerous studies have been published regarding the application of these methods for the discrimination of rice origin such as the near-infrared technique [14,15,16], Raman spectroscopy [17,18], ^1^H-NMR spectroscopy [19,20,21], multielement analysis [10,22,23,24,25], and isotope ratio mass spectrometry (IRMS) [26,27,28,29,30,31]. All methods have been successfully used to identify the geographical origin of various products.

Isotope ratio mass spectrometry (IRMS) is among the three most widely used methods for geographical origin discrimination and relies on the analysis of the isotope ratio of elements (*δ*^13^C, *δ*^15^N, *δ*^34^S, *δ*^18^O, and *δ*^2^H) [26]. Therefore, it has been extensively used for the discrimination of the geographical origin of rice. Chung et al. (2016) [5] tried to distinguish the geographical origin of the same rice varieties from three different Asian countries (Korea, China, and the Philippines) using isotope ratio mass spectrometry (IRMS) to analyze C, N, O, and S stable isotope ratios. The results showed that *δ*^15^Ν and *δ*^34^S values are affected by geographical origin more than *δ*^13^C values. Kukusamude and Konsgri (2017) [32] examined C, N, and O stable isotope ratios in two different varieties of rice (Thai jasmine and Sungyod rice) cultivated in two different regions of Thailand, and the results showed that all studied isotopes are significantly different between the two rice varieties at a 95% confidence interval, probably due to their different geographical origin and different water resource used for rice cultivation. Suzuki et al. (2008) [13] used stable isotope analysis (*δ*^13^C and *δ*^15^N) for the discrimination of polished rice from 14 different cultivation regions of Australia, Japan, and the USA and concluded that *δ*^13^C and *δ*^15^N can be used for the geographical discrimination of polished rice. A pentagonal radar plot based on isotopic compositions was also used to distinguish different areas. Chung et al. (2018) [22] analyzed the isotope ratio of elements (*δ*^13^C, *δ*^15^N, and *δ*^34^S) for the geographical origin discrimination of rice from six Asian countries, and the results revealed that *δ*^34^S could discriminate the geographical origin of rice. The authors also found that *δ*^13^C and *δ*^15^N values alone have weak discriminatory power, while in other [5,13] studies the combination of all isotope ratios could efficiently discriminate the geographical origin of rice. Liu et al. (2019) [23] used stable isotope ratios (*δ*^13^C and *δ*^15^N) to discriminate the geographical origin of rice cultivated in different regions in China and rice imported from Southeast Asia. Results indicated that the origin of rice was discriminated using stable isotope ratio analysis both for samples from different regions in China and samples imported from Southeast Asia.

The aim of this study was to develop isotopic fingerprints of rice samples cultivated in “Agrinio” (Western Greece), “Serres”, and “Chalastra” (Central Macedonia) using stable isotope ratio methodology. In combination with the application of chemometric techniques, the determination of specific isotopic ratios for each cultivation area can be used for discrimination of the geographical origin of rice samples.

## 2. Materials and Methods

### 2.1. Sampling

**Figure 1:**
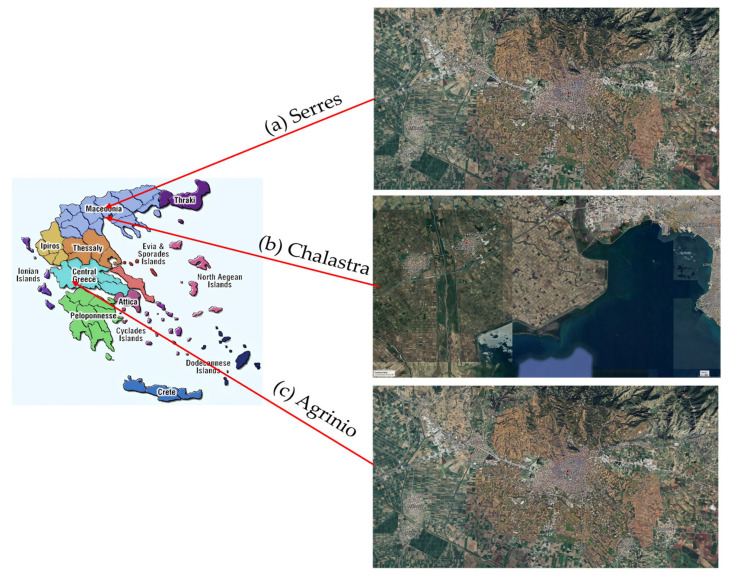
The cultivation areas of the examined rice samples: (**a**) Serres, (**b**) Chalastra, (**c**) Agrinio (Pictures from Google Earth).

Sampling was performed in two harvesting years in each area. The first was in September 2021 and the second in September 2022 for “Agrinio” (Figure 1c) and “Serres” (Figure 1a). In “Chalastra” (Figure 1b) the sampling periods were October 2023 and October 2024. The number of samples was one hundred and twenty (120) from Agrinio, one hundred and sixty (160) from Serres, and ninety (90) from “Chalastra” for both harvesting years. Samples were collected from various producers who follow the traditional cultivation practices. For each field, the sampling was 1 kg/ha. After collection, the samples of each field were mixed and stored at −18 °C for 7 days so that all living components that could have affected our samples were eliminated.

### 2.2. Sample Treatment

**Figure 2:**
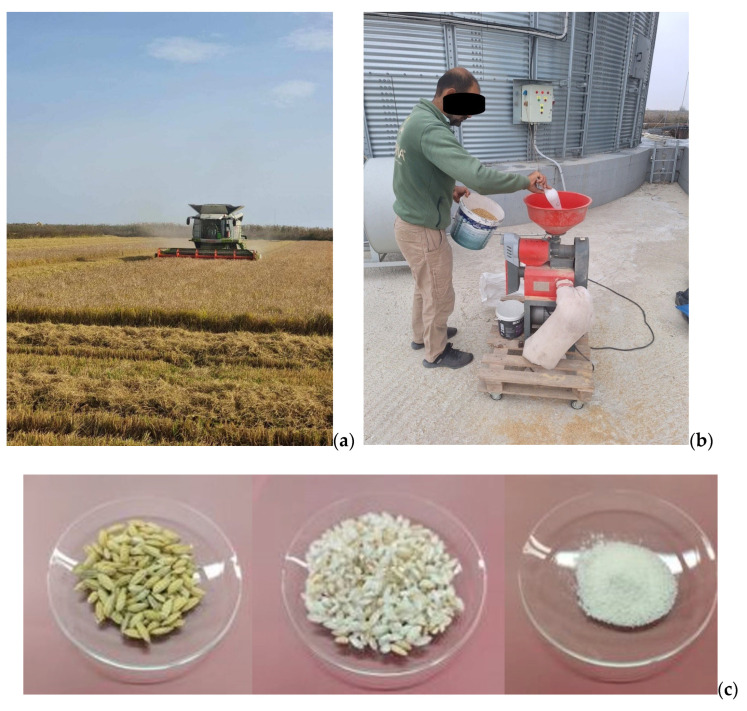
Rice sample harvesting (**a**), rice dehusking (**b**), rice samples before and after pulverization (**c**).

Prior to analysis, 100 gr of each rice sample were unhusked in a semi-industrial machine (see Figure 2b), ground in a mill (pulverisette 11, Fritsch GmbH 93 Milling and Sizing, Idar-Oberstein, Germany) to fine powder, and oven-dried at 60 °C for 48 h. Then, they were stored in falcon tubes and placed in glass desiccators until IRMS analysis (Figure 2).

### 2.3. EA-IRMS Analysis

A portion of the samples of 5 mg was weighed and placed in tin capsules (4 × 4 × 11 mm, Elementar Analysensysteme GmbH, Hanau, Germany) in duplicate and analyzed for C, N, and S isotope ratios, e.g., ^13^C/^12^C, ^15^N/^14^N, and ^34^S/^32^S. For the analysis, a continuous flow system consisting of an Elemental Analyser (Elementar Vario Isotope EL Cube, Elementar Analysensysteme GmbH, Hanau, Germany) interfaced with an Elementar Isoprime 100 Isotope-Ratio Mass Spectrometry (IRMS) instrument (IsoPrime Ltd., Cheadle Hulme, UK) was used. During the analysis, the quantitative combustion of each sample was performed with oxygen added to the helium stream at a temperature of 1150 °C in the elemental analyzer, and the elements C, N, and S bound in the samples were burned to form the following gaseous reaction products: CO_2_, H_2_O, N_2_, NO_X_, SO_2_, and SO_3_. The gaseous products of the combustion were carried into the reduction tube where the NO_x_ and SO_x_ gases were reduced at 850 °C to produce N_2_ and SO_2_. The gases obtained were then introduced into the isotope ratio mass spectrometer.

The results of the isotope ratio analyses were expressed in permille (‰) using the delta *δ* notation and calculated according to the following equation:

(1) *δ* Χ = (R_sample_/R_standard_) − 1

where X is the isotope being studied (e.g., ^13^C, ^15^N, ^34^S), R_sample_ is the isotopic ratio of each measured element in its physical form (e.g., ^13^C/^12^C, ^15^N/^14^N, and ^34^S/^32^S) in the sample, and R_standard_ is the isotopic ratio of the reference material. Firstly, a calibration of the IRMS instrument was carried out using reference materials of known compositions (standard substances). Each reference material was selected according to the isotope that would be determined (C, N, or S). Additionally, an important factor when choosing each standard substance was its composition and the closeness of its isotope ratios to those of the examined samples. The results were normalized to VPDB using ΙAEA-600 (Caffeine, IAEA, Vienna, Austria) and Β2155 (Protein IRMS Standard, Elemental Microanalysis Ltd., Okehampton, UK), with assigned carbon isotope delta values and standard uncertainties (*δ*^13^C_V-PDB_ = −27.77‰ ± 0.043‰ and *δ*^13^C_V-PDB_ = −26.98‰ ± 0.13‰, respectively), to the atmospheric nitrogen (AIR-N_2_) using IAEA-311 (Ammonium sulfate, IAEA, Vienna, Austria) and Β2155 (Protein IRMS Standard, Elemental Microanalysis Ltd., Okehampton, UK), with assigned nitrogen isotope delta values and standard uncertainties (*δ*^15^N_Air_ = 2.05‰ ± 0.04‰ and *δ*^15^N_Air_ = 5.83‰ ± 0.08‰, respectively), and to the VCDT scale using ΙAEA-S1 (Silver Sulfide, IAEA, Vienna, Austria) and Β2155 (Protein IRMS Standard, Elemental Microanalysis Ltd., Okehampton, UK), with assigned sulfur isotope delta values and standard uncertainties (*δ*^34^S_V-CDT_ = −0.3‰ ± 0.03‰ and *δ*^34^S_V-CDT_ = 6.18‰ ± 0.80‰, respectively). The software used for the extraction of results was Vario ISOTOPE cube V4.0.7 and IonVantage 1.7.3.0 for IsoPrime software. The daily routine consisted of each reference material measurement twice at the beginning, after every four sample measurements, and at the end of the analyses. For each sample two repetitions were also performed.

An important factor to consider was that the measured portions of reference materials and samples evolved equivalent amounts of gas. During sample analysis, a quality-control-check sample with B2159 (Sorghum Flour IRMS Standard, Elemental Microanalysis Ltd., Okehampton, UK, with *δ*^13^C_V-PDB_ = −13.78‰, *δ*^15^N_Air_ = 1.58‰, and *δ*^34^S_V-CDT_ = 10.11‰) was analyzed to test the results of our samples [33].

### 2.4. Data Handling and Statistical Analysis

The dataset used in this study consists of the *δ* values *δ*^13^C_V-PDB_, *δ*^15^N_air_, and *δ*^34^S_V-CDT_ of rice samples collected for a two-year period of three different areas (two in Central Macedonia, Greece, namely, “Chalastra” and “Serres”, and one in Western Greece, namely, “Agrinio”). This dataset was examined with descriptive statistics methods like ANOVA and provided the basis for training and testing the machine learning model, i.e., decision tree, used in this study. In brief, the techniques are presented below.

Analysis of Variance (ANOVA) was used in this study to compare the mean *δ* (‰) values to determine whether there are statistically significant differences among them. ANOVA achieves this by partitioning the total variance observed in the data into components attributable to different sources of variation: between-group and within-group variance. The key idea is that if the between-group variance significantly exceeds the within-group variance, the group means are unlikely to be equal. The ratio of these variances is summarized by the F-statistics, which are used to test the null hypothesis that all group means are equal [34]. In addition, Multivariate Analysis of Variance (MANOVA) was used to test for differences between groups for multiple dependent variables simultaneously. Unlike ANOVA, which examines one dependent variable at a time, MANOVA considers the interrelationships among several continuous outcome variables and assesses whether the mean vectors differ across groups. By doing so, it accounts for correlations between dependent variables, providing a more powerful and comprehensive test of group effects.

A decision tree algorithm was selected for this study due to its simplicity, interpretability, and effectiveness in handling both categorical and numerical data [35]. As a supervised, non-parametric learning method, the decision tree recursively partitions the input space into homogeneous subsets based on feature values, constructing a tree-like model where internal nodes represent decision rules, branches represent outcomes of those rules, and leaf nodes correspond to predicted classes or values [36]. In this study, the model was trained on 70% of the data and evaluated on the remaining 30%. Due to its intuitive structure and minimal preprocessing requirements, the decision tree remains a foundational and versatile tool in machine learning, widely applied across domains such as healthcare, finance, and environmental modeling [37].

## 3. Results

### Stable Isotope Results for Rice Samples

The numbers of samples analyzed were 120 rice samples from Agrinio (Tables S1 and S2; Figures S1–S3) and 160 rice samples from Serres (Tables S3 and S4; Figures S4–S6) for two different cultivation periods (2021 and 2022) and 90 rice samples from Chalastra (Tables S5 and S6; Figures S7–S9) (cultivation periods 2023 and 2024). The mean value of *δ*^15^N_AIR_ is 4.64‰ (min value 0.615‰and max value 6.91‰) for the Agrinio area, 5.34‰ (min value 2.97‰and max value 8.46‰) for the Serres area, and 5.90‰ (min value 3.59‰and max value 8.68‰) for the Chalastra area. As shown in Figure S10, the mean value of *δ*^13^C_V-PDB_ is −26.8‰ (min value −29.6‰and max value −25‰) for the Agrinio area, −26.1‰ (min value −28.5‰and max value −25‰) for the Serres area, and −28‰ (min value −28.6‰and max value −26.6‰) for the Chalastra area. The mean value of *δ*^34^S_V-CDT_ is 3.62‰ (min value 1.09‰and max value 6.57‰) for the Agrinio area, −0.903‰ (min value −5.50‰and max value 5.78‰) for the Serres area, and 4.01‰ (min value 0.88‰and max value 8.97‰) for the Chalastra area. The mean values of the examined isotopes are similar except the *δ*^34^S_V-CDT_ of Serres area, mainly due to the fact that geological structure and distance from the sea differentiate *δ*^34^S_V-CDT_ values. Therefore, distribution of the results must be considered.

**Figure 3:**
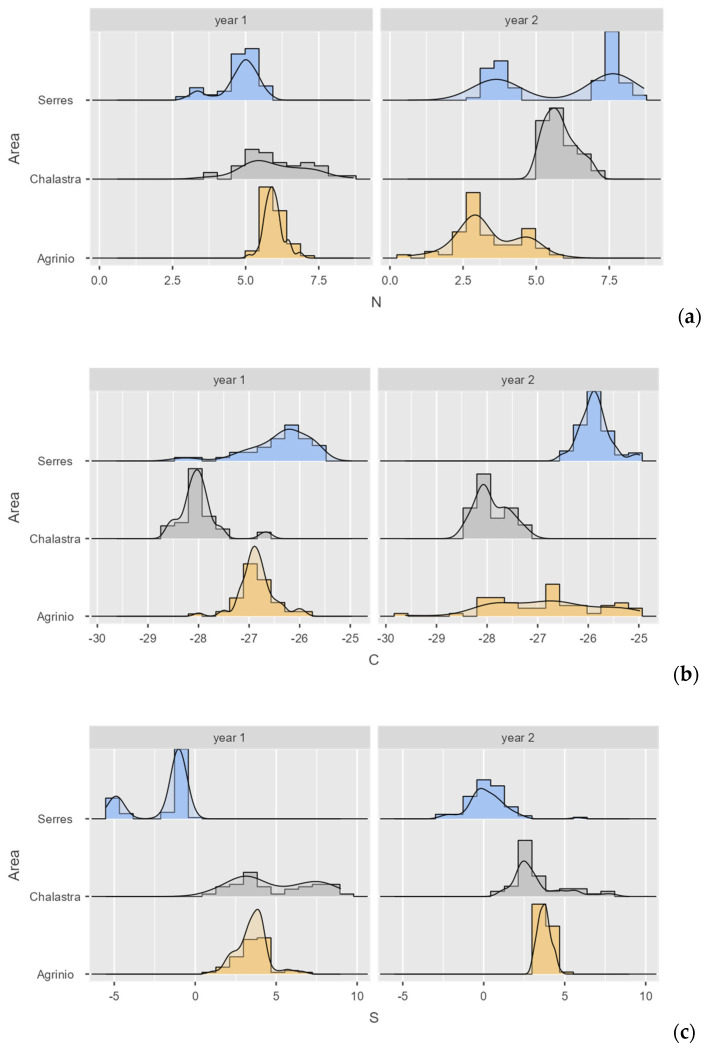
Histograms with density plots for both years of the analyzed isotopes: (**a**) *δ*^15^N_AIR_ (‰), (**b**) *δ*^13^C_V-PDB_ (‰), (**c**) *δ*^34^S_V-CDT_ (‰). Note: The x-labels are simplified forms of the actual variables (e.g., “N” instead of “*δ*^15^N_AIR_ (‰)”).

The descriptive results are presented in Table 1, while Figure 3a–c present the histograms with density plots of the measured *δ* value distributions. Also Table A1 in Appendix A presents the correlation matrix among the examined *δ* values.

The next step in the analysis was to evaluate the effect of the sampling area and sampling period named “Year” in the measured *δ* values. The following Table 2 presents the subsequent results. All tested effects (Area, Year, and their interaction) are statistically significant (*p* < 0.001), indicating meaningful differences in the multivariate outcome measures across areas, years, and their combinations. The magnitude of Pillai’s Trace (Table 3) suggests that there is a very strong and highly significant multivariate effect of “Area” on the combined dependent variables (*δ* values). Also “Area” explains a large portion of variance. In addition, the multivariate effect of “Year” is significant but less so than “Area”. Finally, there is a significant interaction effect of “Year” and “Area”, meaning that the influence of “Area” on the dependent variables (*δ* values) differs depending on the “Year” (or vice versa). In brief “Area” has the strongest overall effect, followed by the “interaction”, then “Year”. The above results suggest that the pattern of measurements differs significantly across different areas, changes somewhat over years, and that the yearly changes are not uniform across areas.

The results indicate that the mean values of all the studied isotopes are statistically different for the examined areas for the periods examined. Nevertheless, the above data are based only on two different cultivation periods and thus need further examination. Since stable isotope ratios differ along with areas, these discrepancies correspond to the distinct environmental and geological factors that formulate each area’s unique isotopic fingerprint. As a result, this unique combination of regional characteristics may influence the classification of products based on their specific stable isotope ratio.

Then, the measured data of *δ*^15^N_AIR_ (‰), *δ*^13^C_V-PDB_ (‰), and *δ*^34^S_V-CDT_ (‰) for the two sampling periods were used in the decision tree algorithm to consider whether the three sampling areas can be discriminated. The decision tree algorithm was developed by using Jamovi statistical Program (ver 2.6.26.0) in order to classify a three-class categorical variable representing the regions “Agrinio”, “Serres”, and “Chalastra”. Even though different classification approaches were evaluated (see Table A2 in Appendix A), the classification tree, as already mentioned in data handing section, was selected to be presented here due to its simplicity and interpretability.

**Figure 4:**
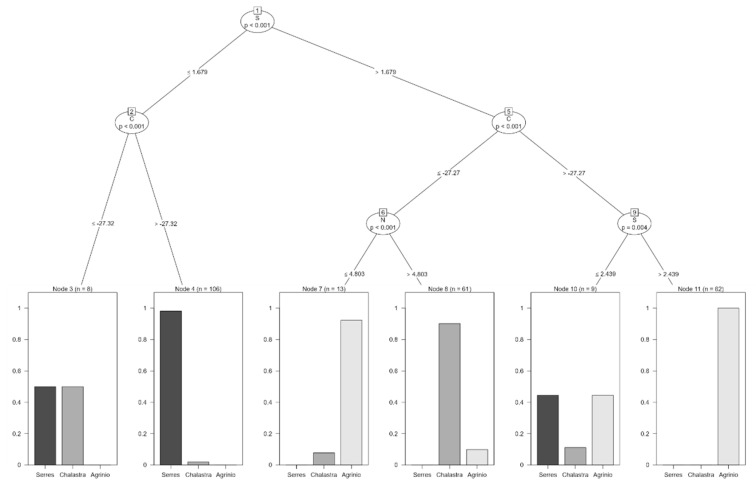
Decision tree plot with training set for both years and all areas of the analyzed isotopes: (a) *δ*^15^N_AIR_ (‰), (b) *δ*^13^C_V-PDB_ (‰), (c) *δ*^34^S_V-CDT_ (‰) (labeled N (a), C (b), and S (c) for simplicity).

The dataset included 259 observations (70% of the total samples) and three predictor *δ* values (*δ*^15^N_AIR_ (‰), *δ*^13^C_V-PDB_ (‰), and *δ*^34^S_V-CDT_ (‰). The model’s simplicity and interpretability make it suitable for domain experts. The decision tree model is depicted in Figure 4. The decision tree is based on continuous *δ* values (labeled N (a), C (b) and S (c) for simplicity). The classification tree uses a hierarchical set of threshold-based decisions on the variables N, C, and S to classify each observation into one of the three regions: “Agrinio”, “Serres”, or “Chalastra”. Each terminal node (leaf) represents a final classification, with the distribution of regions shown as bar plots. The model suggests that specific combinations of the variables are strong predictors of each geographic region.

In the next part of the analysis, a decision tree algorithm was applied using the rpart 4.1.24 (Recursive Partitioning) package in R, which enhances interpretability compared to the previous plot. To ensure the model’s reliability, a 10-fold cross-validation procedure was implemented using the caret package. This approach partitions the dataset into ten equal parts, trains the model on nine parts, and tests on the remaining part—repeating the process ten times. The model’s performance was assessed based on accuracy (0.895) and Kappa (0.837) statistics across folds. The complexity parameter (cp) was tuned during cross-validation. This parameter controls the tree’s growth by preventing unnecessary splits that do not meaningfully reduce classification error. The optimal value (cp = 0.052) was selected based on the highest average accuracy across folds (90%). The final model was trained on the full dataset using the optimal cp and visualized to interpret the decision structure.

The resulting tree clearly highlights key decision thresholds, particularly involving variables *δ*^13^C_V-PDB_ (‰) and *δ*^34^S_V-CDT_ (‰), while variable *δ*^15^N_AIR_ (‰) was found to have no influence on classification. According to “rpart” variable importance results, *δ*^13^C_V-PDB_ = 100.00, *δ*^34^S_V-CDT_ = 94.11%, *δ*^15^N_AIR_ = 16.80%, and sampling period “Year” = 0.00%.

The first and most influential split occurs at *δ*^34^S_V-CDT_ < 1.7‰ When this condition is met, the model confidently predicts “Serres”, with a 95% probability, covering 44% of the total dataset. If *δ*^34^S_V-CDT_ is greater than or equal to 1.7‰, the next split occurs at *δ*^13^C_V-PDB_ < −28‰. For values of *δ*^13^C_V-PDB_ above this threshold, the predicted class is “Agrinio”, with 93% probability, representing 27% of the data. For lower values of *δ*^13^C_V-PDB_, the observation is most likely “Chalastra” (81% probability, 26% of data).

**Figure 5:**
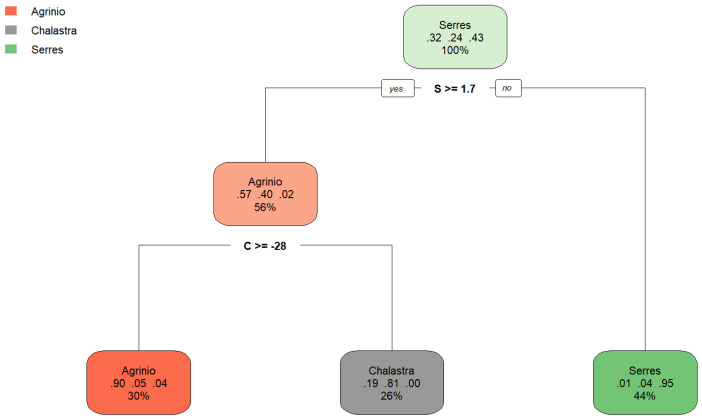
The rpart plot after a 10-fold cross validation procedure for all areas and sampling periods of the analyzed isotopes: (a) *δ*^15^NAIR (‰) and (b) *δ*^13^CV-PDB (‰) (labeled N, C, and S for simplicity).

Figure 5 presents the plot of the classification tree model after the 10-fold cross validation procedure. The model classifies observations into one of three classes: “Serres” (green), “Chalastra” (gray), or “Agrinio” (orange). Furthermore, each node displays the probability distribution of each class at that node and the percentage of the total data that falls into that node (for example, the gray node in the third row can be interpreted as follows: if *δ*^34^S_V-CDT_ ≥ 1.7 and *δ*^13^C_V-PDB_ < −28, the prediction is “Chalastra” with probability 81%, while the probability of belonging to “Agrinio” is 19% and to “Serres” is 0%, and this covers 26% of the data.

The performance of the decision tree model was evaluated using both the training and test datasets (see Table 4 and Table 5), demonstrating high predictive power, generalizability, and balanced performance across all three geographical classes, with particularly strong results for “Serres” and “Chalastra” and slightly lower, yet still strong, performance for “Agrinio”. On the training set, the model achieved an overall accuracy of 93.1%, with a 95% confidence interval ranging from 89.2% to 95.8%, and a Kappa statistic of 0.892, indicating almost perfect agreement between predicted and actual classes. The confusion matrix reveals that most misclassifications occurred within the Serres class (seven instances misclassified as “Chalastra” and four as “Agrinio”), while “Chalastra” and “Agrinio” were generally predicted with high precision. On the test set, the model maintained high performance with an accuracy of 91.9% and a 95% confidence interval between 85.2% and 96.2%. The Kappa value of 0.874 again reflects substantial agreement. The confusion matrix for the test set shows that “Serres” was classified with particularly high sensitivity (0.979), and 47 out of 48 true “Serres” cases were correctly identified. “Chalastra” also showed strong results, with 24 out of 27 cases correctly classified and only minor misclassification into “Agrinio”. “Agrinio” was slightly more challenging, with a sensitivity of 0.861 and three misclassified cases (two predicted as “Chalastra” and one as “Serres”).

Looking at class-specific metrics from the test set, the model exhibited high F1-scores across all three classes: 0.940 for “Serres”, 0.923 for “Chalastra”, and 0.886 for “Agrinio”. The balanced accuracy was also excellent, being 0.950 for “Serres”, 0.938 for “Chalastra”, and 0.911 for “Agrinio”, showing that the model performs well across classes despite differences in class prevalence. The positive predictive values (precision) were also high—over 90% for all three areas—suggesting the model is reliable not only in detecting but also in accurately predicting each class.

It must be noted that a variable indicating the different sampling year was used in the analysis as an independent variable that affects the classification of the samples. Nevertheless, this “sampling year” variable is not present in the classification rules derived by the decision tree algorithm. This apparent contradiction can be attributed to several underlying factors. A first explanation approach involves the fact that this is possible due to the differences between the two years incorporated in *δ*‰ values, allowing the decision tree to detect patterns indirectly associated with specific years. In addition, the number of observations was almost evenly distributed across the two years, avoiding biases toward the characteristics of the dominant year. Decision tree models, such as the one used in this analysis, are particularly sensitive to such underlying patterns and may reconstruct year effects through interactions with other variables, even when the year itself is not explicitly modeled. Therefore, the observed year-related effects suggest that year-to-year variation did not play a substantial role in shaping the outcomes. Further investigations using more sampling years could shed some more light on this.

Stable isotope ratios have been extensively used for the discrimination of the geographical origin of rice in various regions [5,13,22,23,25,38,39,40,41,42,43,44]. Generally, *δ*^13^C_V-PDB_ values are influenced by the photosynthesis pathway of carbon dioxide fixation and environmental factors such as average temperature, solar intensity, humidity, and irrigation.

Also, rice *δ*^13^C values are lower when plants grow in warm and humid environments due to greater CO_2_ transfer to plant tissues through stomata opening. Furthermore, altitude is related to *δ*^13^C values, with high altitudes associated with higher *δ*^13^C values. In the present study, stable isotope ratio analysis was performed for the discrimination of the geographical origin of rice samples from three different areas. *δ*^13^C_V-PDB_ mean values for the three areas and different sampling periods ranged from −28‰ in Chalastra to −26.8‰ in Agrinio and −26.1‰ in Serres. This can be attributed to the fact that Agrinio and Serres are at a higher altitude, while Chalastra is at the lowest. All regions have a temperate climate that is characterized by high temperatures and moderate humidity. Nevertheless, rainfall seems to be different between the areas, with the Agrinio region exhibiting high annual rainfall in contrast with the Serres and Chalastra regions. Chung et al. (2016) [5] reported *δ*^13^C values that ranged from −28.93‰ to −27.51‰ for the three areas examined. The results, which are in agreement with our study, may be associated with differences in climatic environmental factors of each cultivation area. In another study [43], the values of *δ*^13^C for rice samples from eight producing areas in Asia ranged from −28.68‰ to −26.65‰, being significantly different between different areas mainly due to different temperatures but also different farming practices like irrigation.

Nitrogen values are influenced by agricultural practices, the type and amount of fertilizer soil type, and conditions like the available organic nitrogen in soil and temperature [43]. In the present study, *δ*^15^NAIR mean values ranged between 4.64‰ for Agrinio and 5.90‰ for Chalastra for the two sampling periods and were affected by the cultivation practice (type of fertilization). Similarly to another study [5] rice is cultivated in different regions with different fertilization needs in amounts and types that depend on the cultivated soil. The values of *δ*^15^NAIR ranged from 3.58‰ to 6.30‰, and, therefore, this variation in *δ*^15^NAIR values can differentiate the geographical origin of rice that is affected by different type of fertilization.

The isotope ratio of sulfur is affected by the sulphate in the soil (geological structure and organic matter presence) and sulphate in the atmosphere (distance from the sea, sea-spray effect, and anthropogenic emissions). In the present study, mean values of *δ*^34^SV-CDT varied from −0.903‰ in Serres to 4.01‰ in Chalastra for the different sampling periods, probably due to the fact that Agrinio and Chalastra regions are closer to the sea than Serres, which results in elevated *δ*^34^SV-CDT values. Also, Agrinio and Chalastra have lighter types of soil than Serres. In a similar study [43] *δ*^34^SV-CDT values ranged from −1.77‰ to 5.35‰ between different regions, mainly due to the distance from the sea for some regions and secondly because of soil sulfur. Therefore, sulfur isotope analysis can be used for the geographical origin discrimination of rice.

## 4. Conclusions

Stable isotope ratio analysis has been widely used for the geographical discrimination of various agricultural products. One way ANOVA, Multivariate Analysis of Variance (MANOVA), and classification analysis were performed for discrimination of the geographical origin of rice cultivated in three regions in Greece, indicating that the mean values of all the studied isotopes are statistically different between the areas examined for both sampling periods. The decision tree algorithm was used for the discrimination of the three sampling areas and succeeded in accuracy (91.9%), sensitivity (86.1% for Agrinio to 97.9% for Serres), and specificity. Based on these results, geographical origin discrimination for the three areas studied can be achieved.

Nevertheless, future research of more samples from the cultivation areas or other cultivation areas in Greece or abroad and additional cultivation periods could succeed in adequate discrimination.

## Figures and Tables

**Table 1:** Mean values and standard error for all sampling areas for both sampling years.

*δ* (‰)	Area	N	Mean *δ* (‰)	Std. Error	Year
*δ*^15^N_AIR_ (‰)	Agrinio	60	5.95	0.323	2021
	Serres	80	4.79	0.672	2021
	Chalastra	43	6.00	1.09	2023
*δ*^13^C_V-PDB_ (‰)	Agrinio	60	−26.8	0.344	2021
	Serres	80	−26.4	0.628	2021
	Chalastra	43	−28.0	0.387	2023
*δ*^34^S_V-CDT_ (‰)	Agrinio	60	3.51	0.947	2021
	Serres	80	−1.99	1.71	2021
	Chalastra	43	4.86	2.36	2023
*δ*^15^N_AIR_ (‰)	Agrinio	60	3.34	1.08	2022
	Serres	80	5.88	2.02	2022
	Chalastra	47	5.81	0.523	2024
*δ*^13^C_V-PDB_ (‰)	Agrinio	60	−26.8	1.01	2022
	Serres	80	−25.9	0.302	2022
	Chalastra	47	−27.9	0.333	2024
*δ*^34^S_V-CDT_ (‰)	Agrinio	60	3.74	0.391	2022
	Serres	80	0.183	1.21	2022
	Chalastra	47	3.23	1.57	2024

**Table 2:** Univariate test for sampling areas and sampling years.

Univariate Test	Dependent Variable *δ* (‰)	Sum of Squares	df	Mean Square	F Value	Sig.
Area	*δ*^15^N_AIR_ (‰)	82.82	2	41.412	30.33	<0.001
	*δ*^13^C_V-PDB_ (‰)	189.72	2	94.861	295.71	<0.001
	*δ*^34^S_V-CDT_ (‰)	2005.74	2	1002.868	482.84	<0.001
Year	*δ*^15^N_AIR_ (‰)	16.35	1	16.354	11.98	<0.001
	*δ*^13^C_V-PDB_ (‰)	6.12	1	6.117	19.07	<0.001
	*δ*^34^S_V-CDT_ (‰)	35.22	1	35.223	16.96	<0.001
Area x Year	*δ*^15^N_AIR_ (‰)	237.23	2	118.617	86.87	<0.001
	*δ*^13^C_V-PDB_ (‰)	5.39	2	2.697	8.41	<0.001
	*δ*^34^S_V-CDT_ (‰)	214.45	2	107.227	51.63	<0.001
Residuals	*δ*^15^N_AIR_ (‰)	497.03	364	1.365		
	*δ*^13^C_V-PDB_ (‰)	116.77	364	0.321		
	*δ*^34^S_V-CDT_ (‰)	756.04	364	2.077		

**Table 3:** Multivariate test (Pillai’s Trace) for sampling areas and sampling years.

	Value	F Value	DF1	Df2	Sig.
Area	1.122	154.7	6	726	<0.001
Year	0.109	14.7	3	362	<0.001
Area x Year	0.538	44.5	6	726	<0.001

**Table 4:** Overall statistics for the decision tree model.

**Accuracy and kappa for the decision tree model**			
	(A) With training set	Lower	Upper
Accuracy 95% CI	0.931	0.892	0.958
Kappa	0.892		
	(B) With test set	Lower	Upper
Accuracy 95% CI	0.919	0.852	0.962
Kappa	0.874		

**Table 5:** Confusion matrix of the evaluation of the predictions of the decision tree model.

**Prediction evaluation of the decision tree model**			
(A) With training set			
Predicted			
	Serres	Chalastra	Agrinio
Serres	112	7	4
Chalastra	0	55	6
Agrinio	0	1	74
(B) With test set			
Predicted			
	Serres	Chalastra	Agrinio
Serres	47	1	4
Chalastra	0	24	1
Agrinio	1	2	31

## Data Availability

The original contributions presented in the study are included in the article/Supplementary Materials. Further inquiries can be directed to the corresponding authors.

## Supplementary Materials

The following supporting information can be downloaded at: https://www.mdpi.com/article/10.3390/foods14183163/s1, Table S1: Sample dataset from Agrinio for the year 2021; Figure S1: Boxplot diagram from Agrinio for the year 2021; Table S2: Sample dataset from Agrinio for the year 2022; Figure S2: Boxplot diagram from Agrinio for the year 2022; Figure S3: Boxplot diagram from Agrinio for the years 2021 and 2022; Table S3: Sample dataset from Serres for the year 2021; Figure S4: Boxplot diagram, from Serres for the year 2021; Table S4: Sample dataset from Serres for the year 2022; Figure S5: Boxplot diagram, from Serres for the year 2022; Figure S6: Boxplot diagram, from Serres for the years 2021 and 2022; Table S5: Sample dataset from Chalastra for the year 2023; Figure S7: Boxplot diagram from Chalastra for the year 2023; Table S6: Sample dataset from Chalastra for the year 2024; Figure S8: Boxplot diagram from Chalastra for the year 2024; Figure S9: Boxplot diagram from Chalastra for the years 2023 and 2024; Figure S10: Boxplot diagrams of *δ*^13^C (a), *δ*^15^N (b), and *δ*^34^S (c) from Agrinio, Serres, and Chalastra.

## Appendix A

**Table A1:** Correlation matrix for *δ* values.

		*δ*^15^N_AIR_ (‰)	*δ*^13^C_V-PDB_ (‰)	*δ*^34^S_V-CDT_ (‰)
*δ*^15^N_AIR_ (‰)	Pearson’s r	—		
	df	—		
	*p*-value	—		
*δ*^13^C_V-PDB_ (‰)	Pearson’s r	−0.114	—	
	df	368	—	
	*p*-value	0.029	—	
*δ*^34^S_V-CDT_ (‰)	Pearson’s r	0.073	−0.354	—
	df	368	368	—
	*p*-value	0.163	<0.001	—

**Table A2:** Mean accuracy and Kappa for different classification approaches.

Model	Mean Accuracy	Mean Kappa
Classification Tree (ctree)	0.931	0.892
Recursive Partitioning (rpart)	0.895	0.837
Random Forest (rf)	0.962	0.941
k-Nearest Neighbors (knn)	0.961	0.940
Partial Least Squares (pls)	0.869	0.798
