# Supplementary material for: Stable Isotope Ratio Analysis for the Discrimination of the Geographic Origin of Rice (Oryza sativa L.)
Source: Foods. 2025 Sep 11;14(18):3163. doi: 10.3390/foods14183163 (PMC12469187; doi:10.3390/foods14183163)
Supplement: Supplementary file 1 [file foods-14-03163-s001.zip › foods-3754632-supplementary.pdf]

Supplementary Material

Stable Isotope Ratios Analysis for the Discrimination of the Geographic Origin of Rice (Oryza sativa L.)

Anna-Akrivi Thomatou, Eleni C. Mazarakioti, Anastasios Zotos, Achilleas Kontogeorgos, Angelos Patakas, Athanasios Ladavos

Table S1: Sample data set from Agrinio for the year 2021

Sample number	Sample	$\delta^{15}N_{AIR}$ (‰)	$\delta^{13}C_{V\text{-}PDB}$ (‰)	$\delta^{34}S_{V\text{-}CDT}$ (‰)
1	Agr_kar_1	2,3460	-27,4151	3,5401
2	Agr_kar_2	4,6063	-27,9988	3,9233
3	Agr_kar_3	2,5195	-26,3724	3,6623
4	Agr_kar_4	2,7609	-25,5992	3,8868
5	Agr_kar_5	3,1362	-25,2423	3,8304
6	Agr_kar_6	3,0560	-26,6395	4,1100
7	Agr_kar_7	3,1527	-26,7376	3,5620
8	Agr_kar_8	4,7909	-27,8453	3,4215
9	Agr_kar_9	4,3251	-28,5100	3,5089
10	Agr_kar_10	5,8892	-27,8352	3,4928
11	Agr_kar_11	3,8778	-29,6130	3,7064
12	Agr_kar_12	2,1579	-26,9653	3,7886
13	Agr_kar_13	2,1787	-27,1472	3,4336
14	Agr_kar_14	2,7878	-27,4287	3,6362
15	Agr_kar_15	1,7522	-27,5528	3,4457
16	Agr_kar_16	4,8026	-27,9829	3,4479
17	Agr_kar_17	4,1807	-28,0342	4,6986
18	Agr_kar_18	4,4241	-27,9210	3,9842
19	Agr_kar_19	1,4895	-26,7733	3,5628
20	Agr_kar_20	3,9023	-26,3072	2,9886
21	Agr_kar_21	0,6152	-26,0440	3,8982
22	Agr_kar_22	2,3825	-25,6501	3,3602
23	Agr_kar_23	2,7716	-26,2225	3,9888
24	Agr_kar_24	2,9101	-25,5126	4,3634
25	Agr_kar_25	3,8064	-25,2175	3,8426
26	Agr_kar_26	3,0549	-25,1654	3,9067
27	Agr_kar_27	2,4940	-25,2762	3,6903
28	Agr_kar_28	3,0956	-26,2659	3,9243
29	Agr_kar_29	2,6941	-26,8445	4,1430
30	Agr_kar_30	3,0745	-26,6595	4,3949
31	Agr_kar_31	2,9884	-26,8124	3,8316
32	Agr_kar_32	3,1203	-26,7650	3,2668
33	Agr_kar_33	3,0498	-26,6310	3,1983

34	Agr_kar_34	5,0032	-27,6656	3,6689
35	Agr_kar_35	4,7967	-27,8817	3,5555
36	Agr_kar_36	4,3018	-27,9249	3,0142
37	Agr_kar_37	4,7739	-28,0057	3,7713
38	Agr_kar_38	2,9889	-27,2138	3,5852
39	Agr_kar_39	2,2695	-27,3763	3,7156
40	Agr_kar_40	2,1364	-27,5314	3,4414
41	Agr_kar_41	4,6727	-28,0111	3,6989
42	Agr_kar_42	4,5957	-28,0734	3,9151
43	Agr_kar_43	4,9648	-27,9687	4,1271
44	Agr_kar_44	3,0640	-26,7767	4,4225
45	Agr_kar_45	5,1217	-26,2992	3,3910
46	Agr_kar_46	1,2343	-26,0824	3,8159
47	Agr_kar_47	3,0190	-25,9101	3,1420
48	Agr_kar_48	2,8952	-25,6889	4,1395
49	Agr_kar_49	2,8411	-25,4532	4,4201
50	Agr_kar_50	3,8054	-25,0233	3,3957
51	Agr_kar_51	2,8879	-25,1492	3,8445
52	Agr_kar_52	2,6691	-25,4538	4,4010
53	Agr_kar_53	2,6561	-26,4366	4,1262
54	Agr_kar_54	3,0662	-26,9222	4,1392
55	Agr_kar_55	3,2245	-26,7823	4,3465
56	Agr_kar_56	2,8486	-26,6872	3,7860
57	Agr_kar_57	3,1826	-26,7157	3,1776
58	Agr_kar_58	3,1894	-26,8251	3,1307
59	Agr_kar_59	5,1907	-27,5246	3,1914
60	Agr_kar_60	4,6988	-27,7146	3,3223

Figure S1: Boxplot diagram from Agrinio for the year 2021

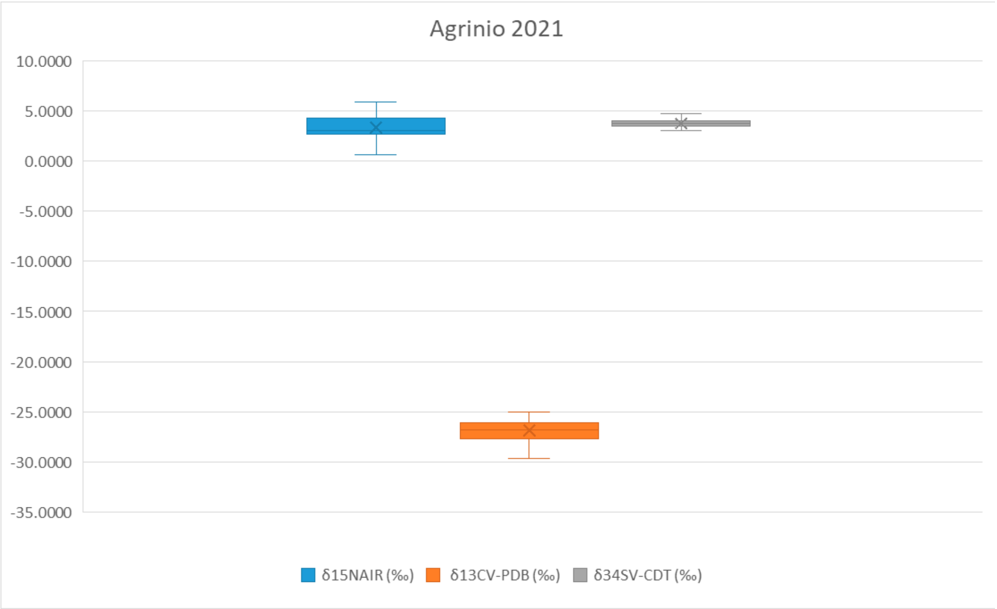

Table S2: Sample data set from Agrinio for the year 2022

Sample number	Sample	$\delta^{15}N_{AIR}$ (‰)	$\delta^{13}C_{V\text{-}PDB}$ (‰)	$\delta^{34}S_{V\text{-}CDT}$ (‰)
1	Agr_kar_1/R2	6,1084	-26,8110	2,7912
2	Agr_kar_2/R2	5,6485	-26,7879	2,9211
3	Agr_kar_3/R2	5,9180	-26,9090	2,5990
4	Agr_kar_4/R2	5,8970	-26,7969	2,7730
5	Agr_kar_5/R2	5,7219	-26,9997	2,9580
6	Agr_kar_6/R2	5,8609	-26,8769	3,2914
7	Agr_kar_7/R2	5,6975	-27,1880	3,3670
8	Agr_kar_8/R2	5,7970	-27,0077	3,3721
9	Agr_kar_9/R2	5,7928	-26,9512	3,1536
10	Agr_kar_10/R2	6,0427	-26,8402	3,2083
11	Agr_kar_11/R2	5,8550	-27,1584	3,9061
12	Agr_kar_12/R2	5,9328	-26,7568	4,0445
13	Agr_kar_13/R2	5,8693	-26,9110	3,5465
14	Agr_kar_14/R2	5,6638	-26,9968	3,3517
15	Agr_kar_15/R2	6,0322	-27,1663	3,9012
16	Agr_kar_16/R2	5,8452	-27,0437	3,7168
17	Agr_kar_17/R2	5,5929	-27,2093	3,2446
18	Agr_kar_18/R2	6,1454	-26,9606	3,4670
19	Agr_kar_19/R2	6,0982	-26,7638	2,0302
20	Agr_kar_20/R2	5,9057	-26,8450	4,0153
21	Agr_kar_21/R2	5,8920	-27,9963	3,8574
22	Agr_kar_22/R2	6,1903	-26,4847	2,4616
23	Agr_kar_23/R2	5,9799	-26,7628	2,2566
24	Agr_kar_24/R2	6,1307	-26,7051	1,8148
25	Agr_kar_25/R2	5,8309	-26,9521	3,6960
26	Agr_kar_26/R2	6,1521	-26,9048	4,1199
27	Agr_kar_27/R2	6,0733	-26,7569	5,5408
28	Agr_kar_28/R2	6,2439	-27,0178	4,1127
29	Agr_kar_29/R2	6,4514	-26,9735	3,9773
30	Agr_kar_30/R2	5,6952	-27,2152	6,5721
31	Agr_kar_31/R2	6,4601	-26,6238	3,8952
32	Agr_kar_32/R2	5,7420	-26,3596	3,8922
33	Agr_kar_33/R2	5,3838	-26,2905	5,8815
34	Agr_kar_34/R2	6,4477	-26,6325	4,1824
35	Agr_kar_35/R2	6,0152	-27,1422	4,0682
36	Agr_kar_36/R2	5,1091	-26,6697	3,7446
37	Agr_kar_37/R2	5,9966	-26,4766	2,3075
38	Agr_kar_38/R2	6,0013	-26,8970	3,3373
39	Agr_kar_39/R2	5,6861	-26,8179	3,5805
40	Agr_kar_40/R2	6,4880	-26,8071	3,9084
41	Agr_kar_41/R2	6,7966	-26,6866	3,5366
42	Agr_kar_42/R2	5,9131	-26,9764	1,9396
43	Agr_kar_43/R2	6,3806	-26,6716	2,2582
44	Agr_kar_44/R2	5,6385	-26,9578	2,0645

45	Agr_kar_45/R2	6,5316	-26,0066	3,9502
46	Agr_kar_46/R2	6,1480	-27,4620	3,5477
47	Agr_kar_47/R2	5,7743	-27,1377	2,4799
48	Agr_kar_48/R2	5,6119	-26,8904	3,4224
49	Agr_kar_49/R2	5,8129	-26,8449	3,4139
50	Agr_kar_50/R2	6,0598	-26,5655	3,9052
51	Agr_kar_51/R2	5,5622	-26,4758	4,0507
52	Agr_kar_52/R2	6,9051	-26,3359	2,9892
53	Agr_kar_53/R2	6,0477	-26,0730	4,1719
54	Agr_kar_54/R2	5,9099	-26,9493	4,2657
55	Agr_kar_55/R2	5,4320	-25,9220	3,1668
56	Agr_kar_56/R2	5,8017	-27,1410	4,1871
57	Agr_kar_57/R2	5,7652	-27,0226	1,0900
58	Agr_kar_58/R2	6,0379	-26,8799	4,0720
59	Agr_kar_59/R2	5,6467	-26,7612	4,3033
60	Agr_kar_60/R2	5,9288	-27,5304	4,8895

Figure S2: Boxplot diagram from Agrinio for the year 2022

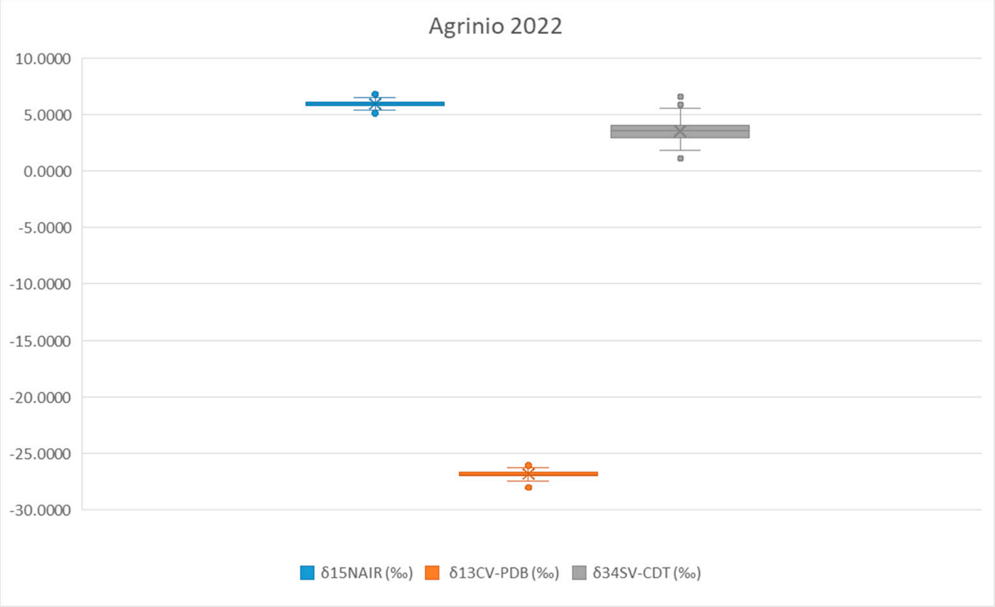

Figure S3: Boxplot diagram from Agrinio for the years 2021 and 2022

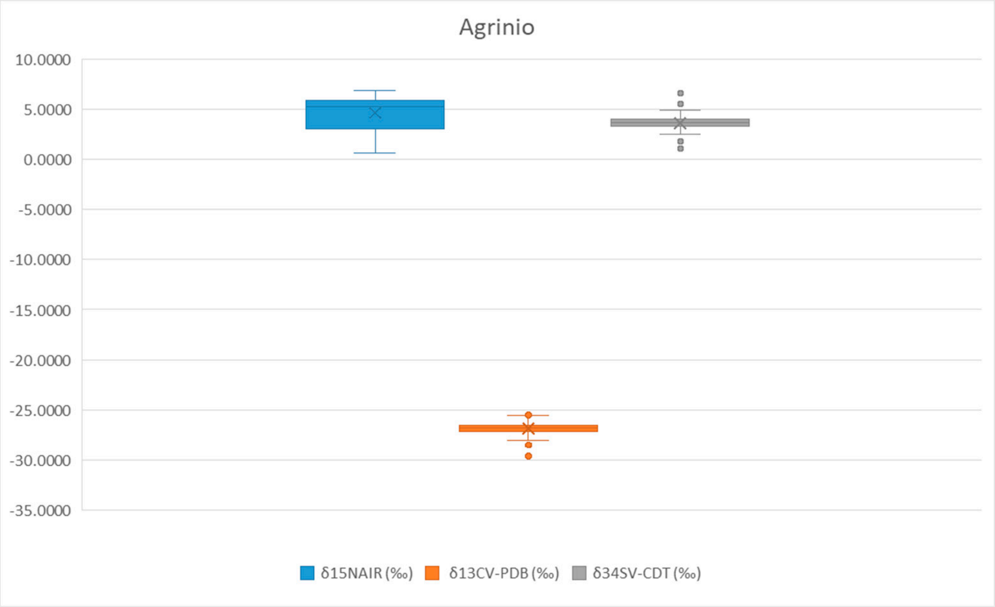

Table S3: Sample data set from Serres for the year 2021

Sample number	Sample	$\delta^{15}N_{AIR}$ (‰)	$\delta^{13}C_{V\text{-}PDB}$ (‰)	$\delta^{34}S_{V\text{-}CDT}$ (‰)
1	Pro_1	5,2568	-26,6759	-4,7180
2	Pro_2	4,6527	-28,1904	-4,9391
3	Pro_3	4,9022	-26,2972	-4,8307
4	Pro_4	4,6359	-26,7853	-5,2004
5	Pro_5	4,2404	-26,4111	-4,8106
6	Pro_6	3,4088	-25,8901	-4,9240
7	Pro_7	5,7621	-26,4442	-4,8950
8	Pro_8	5,4015	-26,8357	-4,6314
9	Pro_9	4,7035	-26,9101	-4,5204
10	Pro_10	4,5687	-28,0187	-4,4124
11	Pro_11	4,5426	-28,3177	-5,4523
12	Pro_12	5,0150	-28,4592	-5,1840
13	Pro_13	4,9977	-26,6738	-4,7573
14	Pro_14	4,3469	-26,1443	-4,1951
15	Pro_15	5,4094	-26,2506	-5,0209
16	Pro_16	4,9840	-26,5250	-4,9544
17	Pro_17	4,3800	-26,9268	-5,4973
18	Pro_18	5,0148	-26,7660	-5,0804
19	Pro_19	4,0081	-26,2843	-4,9117
20	Pro_20	3,0144	-26,0911	-4,8042
21	Tyr_1	3,4841	-27,1685	-1,2712
22	Tyr_2	5,4996	-26,4920	-0,9083
23	Tyr_3	4,8829	-27,0215	-0,4932
24	Tyr_4	3,0139	-27,1250	-1,0305
25	Tyr_5	3,2641	-27,5014	-1,9564
26	Tyr_6	5,2621	-26,5321	-0,6613
27	Tyr_7	5,5883	-26,3285	-1,0619
28	Tyr_8	5,8814	-26,4848	-0,6090
29	Tyr_9	3,7816	-26,8823	-0,9536
30	Tyr_10	3,3078	-27,1400	-1,1007
31	Tyr_11	3,3928	-27,3921	-1,9187
32	Tyr_12	5,5302	-26,3168	-1,1669
33	Tyr_13	5,4076	-26,6189	-1,5705
34	Tyr_14	5,5491	-26,6684	-1,1690
35	Tyr_15	3,8774	-26,9325	-0,9534
36	Tyr_16	3,4380	-27,1758	-1,1703
37	Tyr_17	3,3265	-27,1782	-1,1662
38	Tyr_18	5,5555	-26,2212	-0,3427
39	Tyr_19	5,2990	-26,5496	-1,3031
40	Tyr_20	5,8040	-26,5867	-0,4978
41	Ana_1	5,0817	-25,7960	-0,8550
42	Ana_2	4,8415	-25,6933	-0,8517
43	Ana_3	5,1945	-25,7143	-0,7391

44	Ana_4	4,8712	-25,8734	-0,6882
45	Ana_5	4,4568	-25,8957	-1,1055
46	Ana_6	4,5858	-25,6496	-1,0780
47	Ana_7	5,0479	-25,7516	-0,5501
48	Ana_8	4,9796	-25,8861	-1,2440
49	Ana_9	5,0835	-25,6078	-0,6376
50	Ana_10	4,9271	-25,8454	-0,5635
51	Ana_11	4,6172	-25,7921	-0,9893
52	Ana_12	4,5335	-25,6488	-0,8855
53	Ana_13	4,9577	-25,6345	-0,7583
54	Ana_14	5,0839	-25,8481	-1,1160
55	Ana_15	5,2844	-25,6349	-0,7787
56	Ana_16	5,1303	-25,8537	-0,7249
57	Ana_17	4,4262	-25,8171	-1,2467
58	Ana_18	4,6369	-25,6699	-0,7194
59	Ana_19	4,9080	-25,6412	-0,7914
60	Ana_20	5,0737	-25,8932	-1,1738
61	Vam_1	5,1043	-26,1816	-1,1262
62	Vam_2	4,8014	-26,1061	-1,1982
63	Vam_3	5,0235	-26,3310	-1,0679
64	Vam_4	5,3068	-26,1562	-0,9923
65	Vam_5	4,8386	-26,1191	-1,1933
66	Vam_6	5,0757	-26,2428	-1,1628
67	Vam_7	5,4325	-26,1291	-1,1039
68	Vam_8	4,8741	-26,0862	-1,1764
69	Vam_9	5,2815	-26,2696	-1,0549
70	Vam_10	5,3389	-26,2356	-1,1277
71	Vam_11	4,7322	-26,1519	-1,1928
72	Vam_12	4,9519	-26,3268	-1,0740
73	Vam_13	5,4208	-26,1421	-1,0508
74	Vam_14	4,7790	-25,9273	-1,2824
75	Vam_15	5,2508	-26,2457	-1,1946
76	Vam_16	5,1485	-26,2625	-1,0051
77	Vam_17	4,9261	-26,2286	-1,1691
78	Vam_18	5,2458	-26,2454	-1,0697
79	Vam_19	4,8255	-26,3865	-1,0455
80	Vam_20	4,6278	-26,0956	-1,2440

Figure S4: Boxplot diagram, from Serres for the year 2021

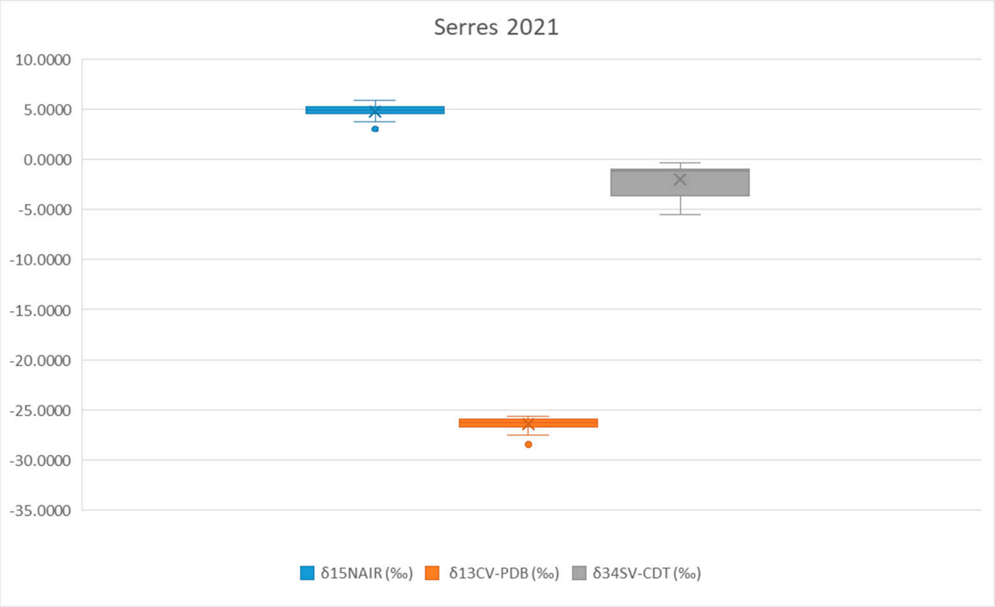

Table S4: Sample data set from Serres for the year 2022

Sample number	Sample	$\delta^{15}N_{AIR}$ (‰)	$\delta^{13}C_{V\text{-}PDB}$ (‰)	$\delta^{34}S_{V\text{-}CDT}$ (‰)
1	Pro_1/R2	7,7899	-26,2390	-0,1866
2	Pro_2/R2	7,7749	-25,9630	0,0272
3	Pro_3/R2	7,6431	-25,4828	0,1548
4	Pro_4/R2	7,5928	-25,7587	0,4697
5	Pro_5/R2	7,3823	-25,9588	0,6911
6	Pro_6/R2	7,3061	-25,9384	0,6816
7	Pro_7/R2	7,7533	-25,6715	0,6858
8	Pro_8/R2	7,3540	-26,0793	0,8525
9	Pro_9/R2	7,3161	-25,9312	0,3829
10	Pro_10/R2	7,5921	-25,8674	0,4280
11	Pro_11/R2	6,8897	-26,1078	2,0445
12	Pro_12/R2	7,5536	-25,8292	-0,8074
13	Pro_13/R2	7,6262	-26,0549	-2,6683
14	Pro_14/R2	7,7707	-25,6943	-0,1646
15	Pro_15/R2	7,2996	-25,7968	1,0514
16	Pro_16/R2	7,6374	-25,9552	0,1456
17	Pro_17/R2	7,4056	-25,9034	-0,1547
18	Pro_18/R2	7,7268	-25,6072	0,6648
19	Pro_19/R2	7,9448	-25,9254	0,0462
20	Pro_20/R2	7,8168	-25,9383	0,2479
21	Tyr_1/R2	7,4711	-26,3112	1,6793
22	Tyr_2/R2	6,9404	-25,7344	1,5640
23	Tyr_3/R2	7,4174	-26,5262	0,4544
24	Tyr_4/R2	8,4626	-25,6926	0,0432
25	Tyr_5/R2	7,7999	-26,1839	1,2854
26	Tyr_6/R2	7,5349	-25,7627	0,5089
27	Tyr_7/R2	8,1706	-25,3877	2,4390
28	Tyr_8/R2	7,7053	-26,1602	0,6679
29	Tyr_9/R2	8,0074	-25,0888	0,8762
30	Tyr_10/R2	7,6546	-25,7938	0,0897
31	Tyr_11/R2	8,3236	-25,9323	-0,3415
32	Tyr_12/R2	7,3422	-26,3375	0,0708
33	Tyr_13/R2	7,1212	-26,1039	0,0265
34	Tyr_14/R2	7,6380	-26,2690	1,1771
35	Tyr_15/R2	6,9327	-25,8559	1,1056
36	Tyr_16/R2	7,3975	-26,1937	-0,2117
37	Tyr_17/R2	7,3645	-25,7127	1,1615
38	Tyr_18/R2	8,0569	-25,1751	1,0107
39	Tyr_19/R2	7,5434	-25,4461	-0,1375
40	Tyr_20/R2	7,9640	-24,9808	0,6489
41	Ana_1/R2	7,9632	-25,7577	0,0820
42	Ana_2/R2	7,7635	-26,1283	-0,2696
43	Ana_3/R2	8,2934	-25,9210	-0,3838

44	Ana_4/R2	7,5474	-26,0485	-0,5808
45	Ana_5/R2	7,8119	-26,0774	-0,3629
46	Ana_6/R2	3,6745	-25,9129	-1,9834
47	Ana_7/R2	3,3792	-25,9590	-1,8882
48	Ana_8/R2	3,4847	-25,7623	-0,5652
49	Ana_9/R2	3,8017	-25,9766	-0,4591
50	Ana_10/R2	3,5968	-25,7012	-0,6740
51	Ana_11/R2	4,0577	-25,7434	-0,0540
52	Ana_12/R2	3,4990	-25,9042	-0,8560
53	Ana_13/R2	3,9212	-26,0932	-2,3621
54	Ana_14/R2	3,8483	-25,5119	-0,3324
55	Ana_15/R2	3,6468	-24,9915	-0,5650
56	Ana_16/R2	3,7305	-25,9805	-0,7337
57	Ana_17/R2	4,1899	-25,9349	-0,3082
58	Ana_18/R2	3,6837	-26,1508	-1,4411
59	Ana_19/R2	4,2176	-25,6234	-0,8610
60	Ana_20/R2	3,8506	-25,8070	-1,2357
61	Vam_1/R2	3,9104	-25,8274	-2,2310
62	Vam_2/R2	3,3379	-25,8762	-1,2685
63	Vam_3/R2	3,5959	-25,7235	-0,4040
64	Vam_4/R2	2,9726	-25,5196	-0,4571
65	Vam_5/R2	3,8401	-25,4667	0,4372
66	Vam_6/R2	4,0685	-25,8484	-0,2506
67	Vam_7/R2	3,5007	-26,5405	1,3490
68	Vam_8/R2	3,6220	-25,6058	1,6568
69	Vam_9/R2	4,1641	-25,8592	1,9109
70	Vam_10/R2	3,8688	-25,9303	1,0385
71	Vam_11/R2	3,6774	-25,8323	1,5982
72	Vam_12/R2	3,8224	-25,7215	0,1970
73	Vam_13/R2	3,2552	-26,0870	-0,4968
74	Vam_14/R2	3,3670	-26,0823	0,5646
75	Vam_15/R2	3,1556	-25,9058	5,7770
76	Vam_16/R2	3,2940	-26,4457	-0,7660
77	Vam_17/R2	3,4459	-25,8170	2,2005
78	Vam_18/R2	3,4402	-26,2062	1,0792
79	Vam_19/R2	3,0955	-25,9072	0,5040
80	Vam_20/R2	3,2241	-26,1908	-0,6747

Figure S5: Boxplot diagram, from Serres for the year 2022

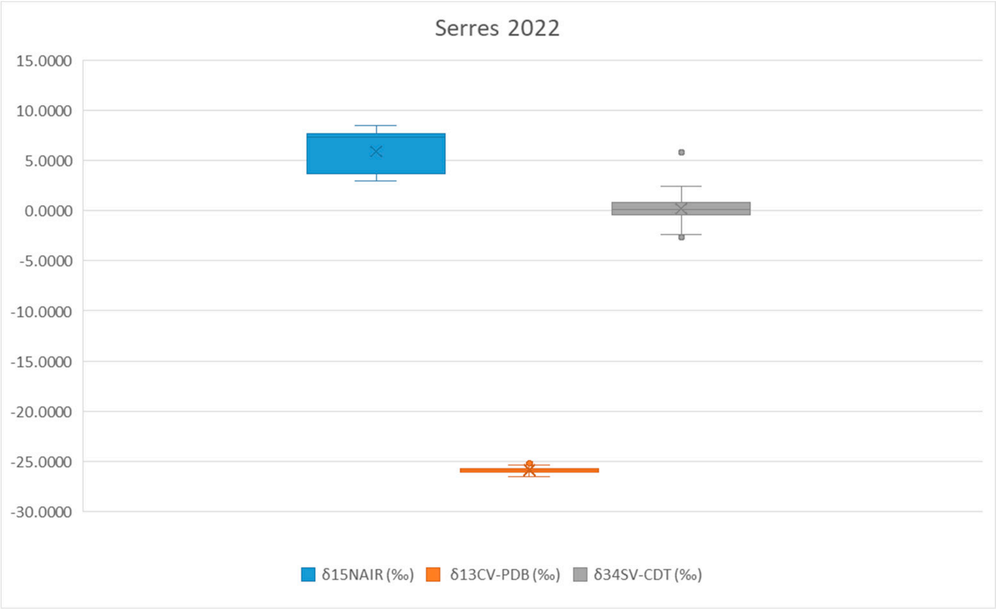

Figure S6: Boxplot diagram, from Serres for the years 2021 and 2022

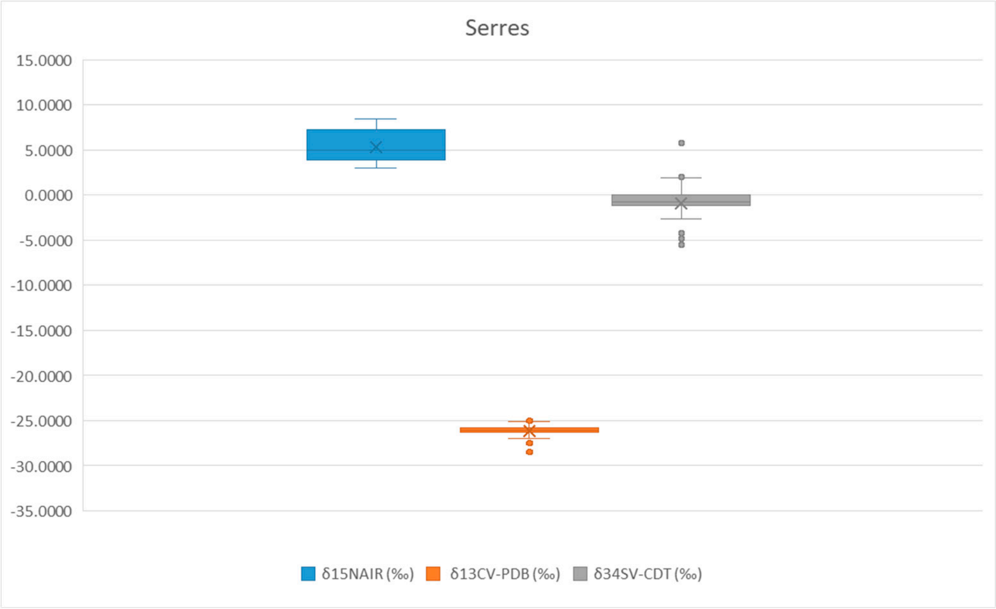

Table S5: Sample data set from Chalastra for the year 2023

Sample number	Sample	$\delta^{15}N_{AIR}$ (‰)	$\delta^{13}C_{V-PDB}$ (‰)	$\delta^{34}S_{V-CDT}$ (‰)
1	RICE_MAL_XAL_1	6,93	-27,54	7,89
2	RICE_MAL_XAL_2	7,49	-28,10	6,93
3	RICE_MAL_XAL_3	7,21	-28,44	7,27
4	RICE_MAL_XAL_4	6,55	-28,54	5,69
5	RICE_MAL_XAL_5	5,49	-28,00	7,29
6	RICE_MAL_XAL_6	5,69	-27,98	8,15
7	RICE_MAL_XAL_7	6,15	-27,97	8,15
8	RICE_MAL_XAL_8	6,17	-28,14	7,15
9	RICE_MAL_XAL_9	5,22	-27,83	4,26
10	RICE_MAL_XAL_10	5,25	-27,78	3,34
11	RICE_MAL_XAL_11	5,14	-28,19	3,16
12	RICE_MAL_XAL_12	5,18	-28,26	4,31
13	RICE_MAL_XAL_13	5,38	-28,18	4,13
14	RICE_MAL_XAL_14	5,45	-28,05	3,25
15	RICE_MAL_XAL_15	6,37	-28,01	6,23
16	RICE_MAL_XAL_16	6,58	-27,96	6,39
17	RICE_MAL_XAL_19	7,70	-28,48	1,64
18	RICE_MAL_XAL_20	7,84	-28,61	1,80
19	RICE_MAL_XAL_21	7,36	-28,12	2,52
20	RICE_MAL_XAL_22	8,68	-28,01	3,54
21	RICE_MAL_XAL_23	5,99	-28,13	2,67
22	RICE_MAL_XAL_24	6,22	-28,12	3,15
23	RICE_MAL_XAL_25	4,84	-28,18	2,74
24	RICE_MAL_XAL_26	5,25	-28,10	2,86
25	RICE_MAL_XAL_27	6,88	-28,21	3,22
26	RICE_MAL_XAL_28	6,64	-28,09	2,14
27	RICE_MAL_XAL_29	6,80	-28,04	6,68
28	RICE_MAL_XAL_30	6,17	-28,09	5,60
29	RICE_MAL_XAL_31	6,12	-28,10	2,36
30	RICE_MAL_XAL_32	5,92	-28,06	2,60
31	RICE_MAL_XAL_33	6,43	-27,86	5,74
32	RICE_MAL_XAL_34	6,04	-27,75	5,15
33	RICE_MAL_XAL_35	5,12	-28,03	2,45
34	RICE_MAL_XAL_36	6,01	-28,32	4,33
35	RICE_MAL_XAL_37	5,39	-28,39	1,56
36	RICE_MAL_XAL_38	5,88	-28,40	2,63
37	RICE_MAL_XAL_39	5,24	-28,03	2,41
38	RICE_MAL_XAL_40	5,63	-28,17	1,30
39	RICE_MAL_XAL_41	5,53	-28,43	2,58
40	RICE_MAL_XAL_42	5,34	-28,11	2,61
41	RICE_MAL_XAL_43	5,80	-28,26	2,65
42	RICE_MAL_XAL_44	5,85	-28,24	2,21
43	RICE_MAL_XAL_45	5.17	-27,93	2.96

Figure S7: Boxplot diagram from Chalastra for the year 2023

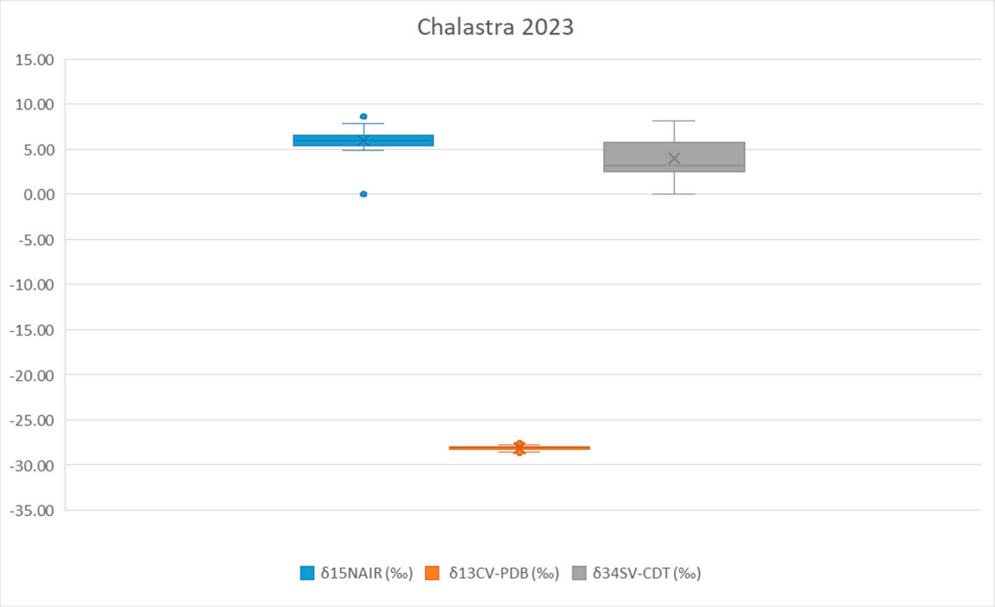

Table S6: Sample data set from Chalastra for the year 2024

Sample number	Sample	$\delta^{15}N_{AIR}$ (‰)	$\delta^{13}C_{V\text{-}PDB}$ (‰)	$\delta^{34}S_{V\text{-}CDT}$ (‰)
1	RICE_MAL_XAL_17	5,79	-28,09	8,09
2	RICE_MAL_XAL_18	5,58	-27,94	8,39
3	RICE_MAL_XAL_46	6,95	-28,02	7,66
4	RICE_MAL_XAL_47	7,18	-27,84	8,97
5	RICE_MAL_XAL_48	4,82	-27,57	5,88
6	RICE_MAL_XAL_49	4,56	-28,58	6,86
7	RICE_MAL_XAL_50	6,65	-27,87	8,22
8	RICE_MAL_XAL_51	6,46	-28,21	7,44
9	RICE_MAL_XAL_52	5,13	-26,73	1,28
10	RICE_MAL_XAL_53	5,66	-26,61	0,88
11	RICE_MAL_XAL_54	4,86	-27,61	3,55
12	RICE_MAL_XAL_55	5,29	-27,84	5,45
13	RICE_MAL_XAL_56	3,89	-28,02	2,00
14	RICE_MAL_XAL_57	3,59	-27,98	3,22
15	RICE_MAL_XAL_58	5,56	-27,82	4,28
16	RICE_MAL_XAL_59	5,52	-27,88	2,89
17	RICE_MAL_XAL_60	7,43	-28,31	3,03
18	RICE_MAL_XAL_61	7,23	-27,96	2,66
19	RICE_MAL_XAL_62	5,22	-28,39	3,13
20	RICE_MAL_XAL_63	5,58	-28,20	3,41
21	RICE_MAL_XAL_64	6,84	-28,24	7,99
22	RICE_MAL_XAL_65	6,90	-28,06	4,76
23	RICE_MAL_XAL_66	5,75	-27,96	2,63
24	RICE_MAL_XAL_67	5,61	-27,78	4,71
25	RICE_MAL_XAL_68	5,00	-27,67	3,10
26	RICE_MAL_XAL_69	5,22	-27,63	5,69
27	RICE_MAL_XAL_70	5,62	-27,65	4,12
28	RICE_MAL_XAL_71	5,36	-27,69	2,91
29	RICE_MAL_XAL_72	5,71	-27,54	7,46
30	RICE_MAL_XAL_73	5,83	-27,59	3,25
31	RICE_MAL_XAL_74	5,36	-27,97	2,01
32	RICE_MAL_XAL_75	5,47	-27,74	3,74
33	RICE_MAL_XAL_76	6,46	-28,05	3,21
34	RICE_MAL_XAL_77	6,45	-28,01	2,36
35	RICE_MAL_XAL_78	6,25	-28,06	3,15
36	RICE_MAL_XAL_79	6,36	-28,03	3,27
37	RICE_MAL_XAL_80	6,29	-28,10	2,43
38	RICE_MAL_XAL_81	5,99	-28,45	3,07
39	RICE_MAL_XAL_82	5,31	-27,56	1,12
40	RICE_MAL_XAL_83	5,11	-27,53	2,35
41	RICE_MAL_XAL_84	5,68	-27,27	1,95
42	RICE_MAL_XAL_85	5,67	-27,35	2,19
43	RICE_MAL_XAL_86	5,77	-27,64	2,36

44	RICE_MAL_XAL_87	5,63	-27,15	2,42
45	RICE_MAL_XAL_88	5,58	-27,44	2,23
46	RICE_MAL_XAL_89	5,41	-27,32	1,18
47	RICE_MAL_XAL_90	5.09	-27,42	2.14

Figure S8: Boxplot diagram from Chalastra for the year 2024

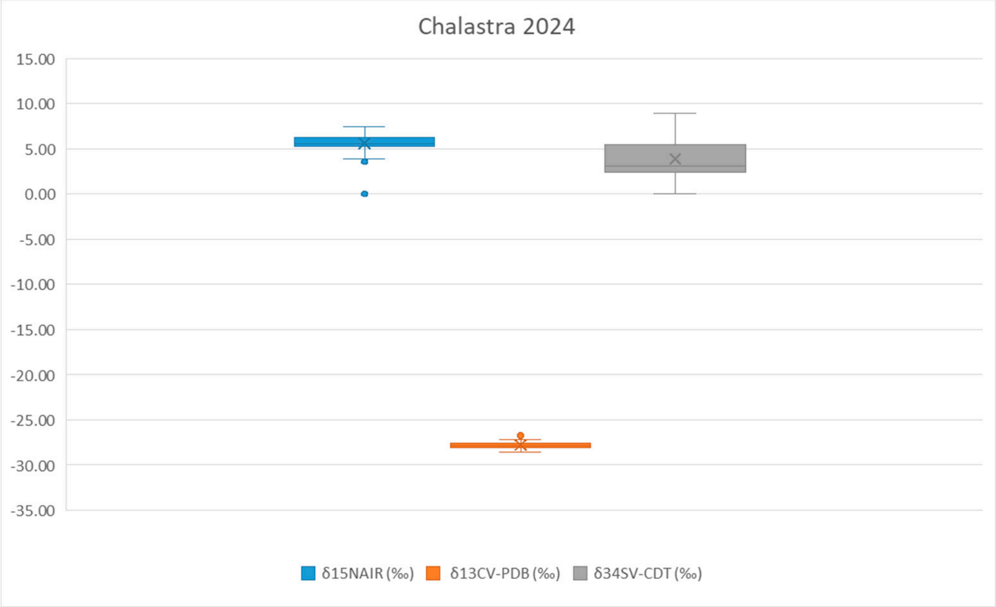

Figure S9: Boxplot diagram from Chalastra for the years 2023 and 2024

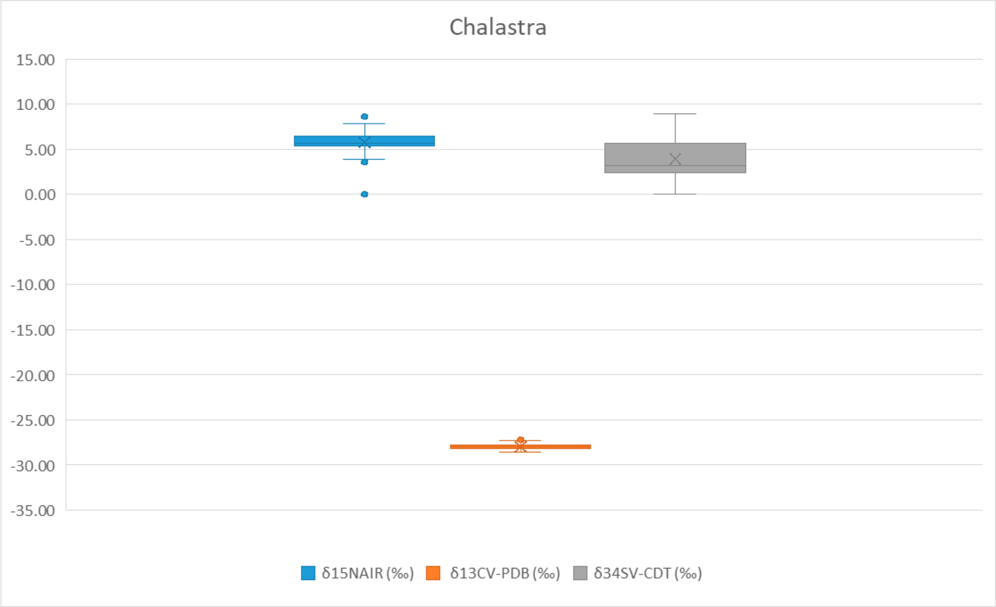

Figure S10: Boxplot diagrams of δ13C (a), δ15N (b), δ34S (c) from Agrinio, Serres and Chalastra

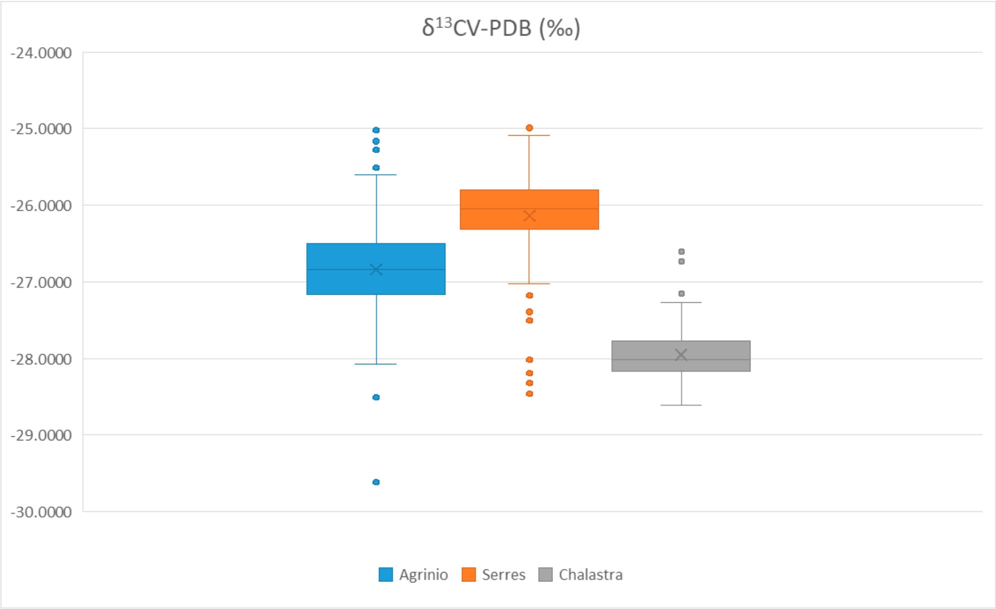

(a)

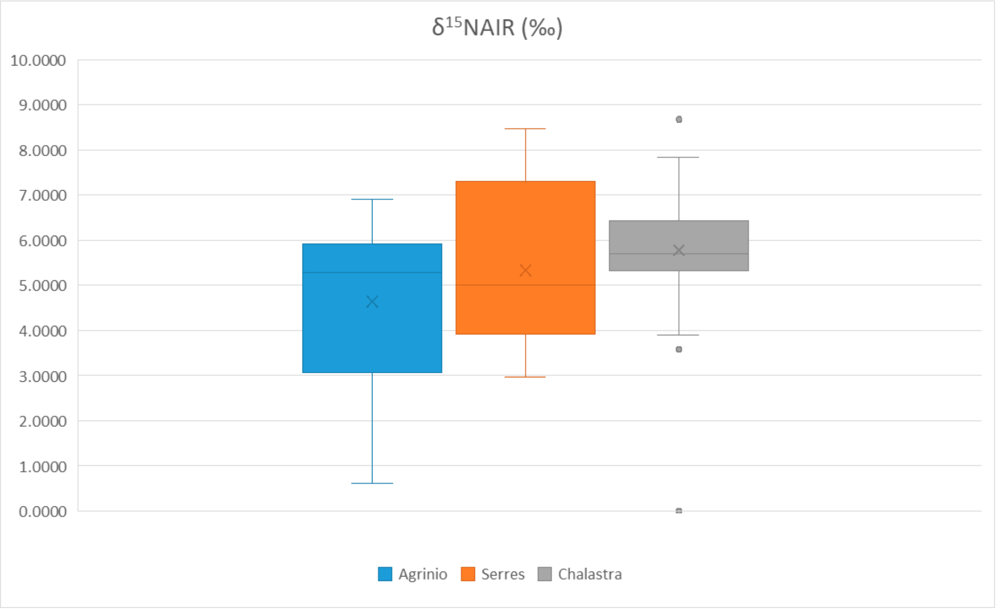

(b)

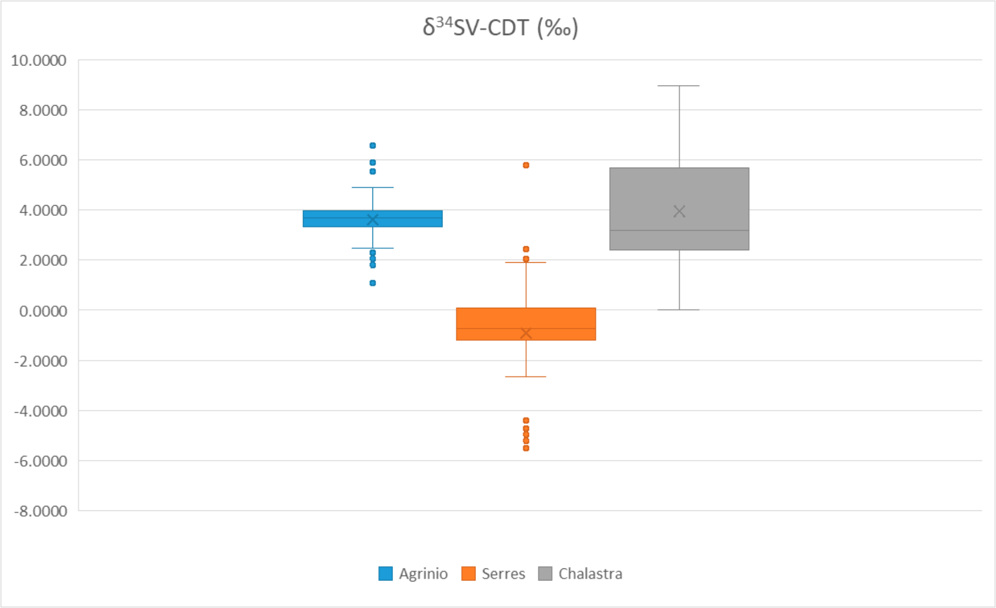
δ34SV-CDT (‰)
10.0000
8.0000
6.0000
4.0000
2.0000
0.0000
-2.0000
-4.0000
-6.0000
-8.0000
Agrinio
Serres
Chalastra
(c)
